# Zero-shot semantic landmark-based visual odometry using foundation models for unstructured planetary exploration

**DOI:** 10.3389/frobt.2026.1819714

**Published:** 2026-07-08

**Authors:** Cristina Pérez-Ramos, Leopoldo Altamirano-Robles, Miguel Chávez-Dagostino

**Affiliations:** 1 Computer Vision Laboratory, Space Science and Technology Department, Instituto Nacional de Astrofísica, Óptica y Electrónica (INAOE), Tonantzintla, Puebla, Mexico; 2 Computer Vision Laboratory, Computer Science Department, Instituto Nacional de Astrofísica, Óptica y Electrónica (INAOE), Tonantzintla, Puebla, Mexico; 3 Astrophysics Department, Instituto Nacional de Astrofísica, Óptica y Electrónica (INAOE), Tonantzintla, Puebla, Mexico

**Keywords:** foundation models, planetary navigation, semantic landmarks, space robotics, visual odometry, zero-shot learning

## Abstract

Precise autonomous navigation on unstructured planetary surfaces is a critical prerequisite for future exploration missions, particularly in GNSS-denied environments such as the Lunar South Pole or Martian deserts. Traditional Visual Odometry (VO) methods, which rely on tracking low-level geometric features (e.g., corners), often fail under the extreme illumination contrast of the Moon or the textural monotony of the Martian regolith. In this work, we present a zero-shot semantic landmark-based visual odometry approach that leverages the generalization capabilities of modern Foundation Models. Our approach uses the Segment Anything Model (SAM) to extract geological landmarks (rocks) and DINOv2 to generate view-invariant semantic descriptors that are matched across frames. We evaluate our pipeline across two distinct domains: a high-fidelity synthetic lunar environment (LuSNAR dataset) to test robustness against extreme lighting, and a real-world Martian analog dataset (Katwijk Beach) to assess sim-to-real transfer. Experimental results show that the proposed approach achieves a decimeter-level trajectory accuracy ( RMSE≈ 0.14 m) on the Martian analog and an RMSE = 1.93 m on the most stable lunar traverse, without any domain-specific fine-tuning. Our results suggest that Foundation-Model-based semantic landmarks are a promising alternative to low-level features for zero-shot VO in planetary-like environments.

## 1 Introduction

As we prepare for a sustained presence in space marked by the Artemis program to the Moon and future missions to Mars, autonomous systems will play an extremely important role. The next era of space exploration will be driven by missions to unexplored and dangerous regions, such as the permanently shadowed craters of the lunar south pole or Martian lava tubes. In this context, the ability of robotic systems to accurately determine their position and orientation within a shared frame of reference is crucial for navigation, exploration, infrastructure deployment, and scientific discovery. Although Earth-based systems benefit from Global Navigation Satellite Systems (GNSS), the Moon and Mars lack GNSS infrastructure, requiring onboard perception-based methods for trajectory estimation. This challenge is intensified by the extreme illumination contrast of the lunar surface and the textural monotony of Martian regolith, both of which severely degrade conventional vision-based methods.

Vision-based navigation techniques are commonly used in environments where GNSS is unavailable or unreliable and can be grouped into three categories. (i) Feature-based visual odometry methods, such as those relying on SIFT (Lowe, 2004), ORB (Rublee et al., 2011), or learned alternatives like SuperPoint (DeTone et al., 2018), to estimate motion by tracking low-level keypoints between consecutive frames. (ii) Map-based localization methods, including image-based place recognition such as Bag of Words (Cummins and Newman, 2008), NetVLAD (Arandjelovic et al., 2016), and AnyLoc (Keetha et al., 2023), and Terrain Relative Navigation (TRN) approaches (Johnson et al., 2007; Vander Hook et al., 2022), that align onboard observations with a previously acquired global or orbital map. Crater-based and shadow-based variants, such as LunarNav (Daftry et al., 2023) and ShadowNav (Atha et al., 2024) belong to this group. (iii) Semantic and object-based methods (Ankenbauer et al., 2023; Ramos et al., 2024) attempt to associate observations through higher-level entities, such as objects with predefined classes, to mitigate perceptual aliasing in repetitive scenes.

Each of these families presents specific limitations when deployed in unstructured planetary terrain. Feature-based VO methods are sensitive to the lack of gradient information in deeply shadowed regions and to the textural repetition of the regolith, which leads to keypoint drift and tracking failure. Map-based methods depend on the availability of high-resolution prior maps and on a sensing modality consistent with the orbital data, and they typically report accuracies in the range of several meters, limited by the resolution of satellite imagery. Object-based methods such as those built on YOLO (You Only Look Once; Redmon and Farhadi, 2018) are restricted to predefined object classes and require domain-specific labeled datasets, which are scarce for planetary surfaces. Hybrid strategies used by past rover missions, including wheel odometry, IMU integration, celestial navigation, and periodic correlation with orbital imagery (Maimone et al., 2006; Parker et al., 2010), are effective for short distances but accumulate unbounded drift over long traverses.

To avoid both relying on previous orbital maps and the cost of acquiring large-scale labeled planetary datasets, recent work has begun to take advantage of the so-called Foundation Models. These large-scale vision models, pre-trained on massive datasets, exhibit strong zero-shot generalization to unseen domains. We present a visual odometry approach that integrates the Segment Anything Model (SAM; Kirillov et al., 2023) to enhance detection of rock-like geological landmarks under extreme lunar illumination and Martian shadows. Furthermore, to ensure robust data association across large changes in perspective, we use DINOv2 (Oquab et al., 2023), a self-supervised vision transformer, to generate semantic descriptors that are significantly more discriminative than traditional SIFT or ORB features and robust to large viewpoint changes.

Our contributions include:

(i) Provide evidence that the foundational models Segment Anything Model (SAM; Kirillov et al., 2023) and DIstillation of knowledge with NO labels v2 (DINOv2; Oquab et al., 2023) exhibit sufficient zero-shot generalization to detect and describe geological landmarks on lunar and Martian terrains, avoiding the need for large-scale labeled planetary datasets.
(ii) Design a zero-shot semantic-landmark-based visual odometry pipeline that combines open-set segmentation, adaptive geometric filtering and foundation model descriptors for stable inter-frame data association.
(iii) Validate experimentally our approach on a high-fidelity synthetic lunar dataset (LuSNAR) and a real-world Martian analog dataset (Katwijk Beach), reporting trajectory accuracy without any domain-specific fine-tuning and analyzing the conditions under which semantic landmarks remain stable across viewpoints.

## 2 Methodology

**FIGURE 1:**
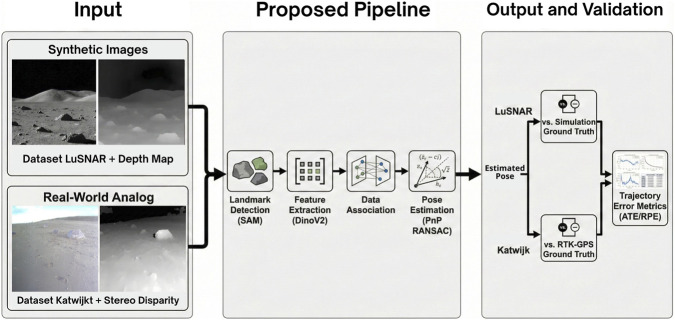
Overview of the pipeline and validation strategy designed to operate in different domains. The algorithm accepts monocular RGB inputs from both synthetic lunar environments and real planetary analogs, in this case LuSNAR and Katwijk, respectively. The main modules (SAM-based detection, DINOv2 feature extraction, and robust tracking) remain invariant. Scale is recovered using either aligned depth maps or stereo disparity, allowing trajectory evaluation with respect to ground truth against the simulated terrain and RTK-GPS data, respectively.

We propose a modular Visual Odometry system designed to operate in unstructured planetary environments, leveraging semantic understanding of foundation models. Unlike traditional approaches that track low-level features (e.g., ORB (Rublee et al., 2011) and SIFT (Lowe, 2004)), our approach tracks high-level geological instances, in this case, rocks. The method (Figure 1) consists of five sequential stages: (1) Data Preprocessing, to standardize multi-domain inputs; (2) Zero-Shot Landmark Proposal, leveraging SAM to segment geological features; (3) Deep Feature Extraction, using DINOv2 to encode semantic descriptors; (4) Data Association, which links landmarks across frames; and (5) Motion Estimation, which computes the rover’s trajectory using robust geometric solvers. Below we describe each stage.

### 2.1 Data representation and preprocessing

The framework takes as input a sequence of monocular RGB images $I_{t}$ and per-frame depth maps $D_{t}$ . We define the input at time t as $F_{t}=\left\{I_{t},D_{t}\right\}$ . The RGB stream feeds the perception frontend (SAM segmentation and DINOv2 description), while the depth map $D_{t}$ provides the metric scale used by the geometric backend. The source of $D_{t}$ depends on the source domain:

1. Synthetic Lunar Domain (LuSNAR): $I_{t}$ is generated from a rendered Unreal-Engine-based photorealistic simulation of the lunar south pole (Liu et al., 2024). The $D_{t}$ depth maps are obtained directly from the simulator’s Z-buffer providing a depth ground truth for validation.
2. Analog Martian Domain (Katwijk): $I_{t}$ is taken from the Katwijk Beach dataset, which was captured by a rover in an unstructured sandy beach environment (Hewitt et al., 2018). Here, the $D_{t}$ depth maps are obtained from stereo disparity, introducing real sensor noise.

### 2.2 Zero-Shot Landmark Proposal

In order to detect stable landmarks without training on specific planetary datasets, we use the Segment Anything Model. We employ Vision Transformer Base (ViT-B) architecture for mask generation, with the challenge of planetary terrain being to distinguish stable rocks from regolith textures, for which we implement a geometric filtering strategy to reject unstable regions.

**FIGURE 2:**
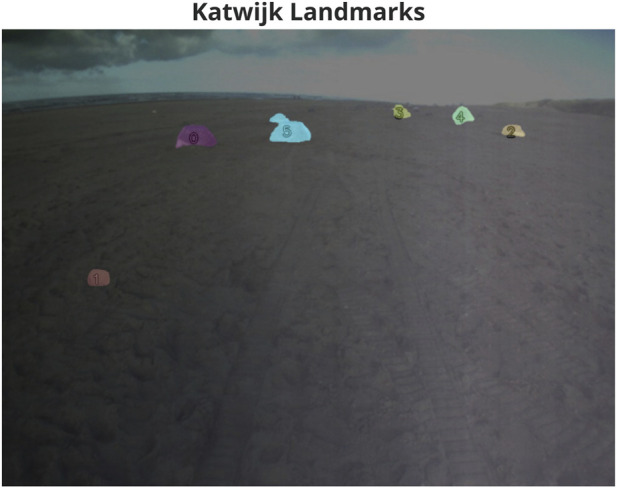
This figure illustrates the segmentation results on the Katwijk dataset (Martian analog), effectively isolating the geological features from the sandy terrain under diffuse lighting. Note that the model operates in a zero-shot approach, utilizing the same pre-trained weights for both scenarios.

For each image $I_{t}$ , SAM generates a set of binary masks $M_{t}=\left\{m_{1},m_{2},\ldots ,m_{k}\right\}$ . We filter the masks according to surface area and solidity, keeping only masks with an area $A\in \left[A_{\min},A_{\max}\right]$ and solidity S>0.85 . This guarantees that only regions with consistent segmentation are retained, as illustrated in Figure 2. For each valid mask $m_{i}$ , we find its geometric centroid $u_{i}=\left(u,v\right)$ in pixel coordinates, which will serve as the 2D keypoint, denoted as $p_{i}^{2D}$ .

### 2.3 Deep Feature Extraction

Traditional descriptors (e.g., BRIEF; Calonder et al., 2010) lack semantic robustness in the context of extreme changes found in environments such as those of the Moon and Mars. We employ DINOv2 ViT-S/14 (Oquab et al., 2023), a self-supervised vision transformer, to encode the semantic identity of each rock.

For each detected landmark $m_{i}$ , we extract a region of interest around the bounding box of the mask, resizing the original image $I_{t}$ to 224×224 pixels. The image region is passed through the DINOv2 encoder to obtain a feature vector $f_{i}\in R^{384}$ from the [CLS] token, which serves as a global semantic summary of the input image patch. The $f_{i}$ vector encodes a high-level semantic representation of the rock, enabling robust re-identification that is invariant to changes in viewpoint and local variations in lighting.

### 2.4 Data association and tracking

Data association is formulated as a linear sum assignment problem. We construct a cost matrix C between the set of tracks T from frame t−1 and the new detections D in frame t . The cost $C_{ij}$ is defined by the cosine dissimilarity between their feature vectors, as shown in Equation 1:

$$\;C_{ij}=1-\frac{f_{T_{i}}\cdot f_{D_{j}}}{\left\|f_{T_{i}}\|\|f_{D_{j}}\right\|}$$ (1)

We use the Hungarian algorithm (Kuhn, 2005) to find the optimal assignment that minimizes the total cost, obtaining pairs of correspondences (i,j) . Associations whose cost exceeds a threshold $C_{ij}>match$ are discarded to avoid false positives.

### 2.5 Motion Estimation

After establishing the correspondences, the rover’s egomotion is estimated by solving the Perspective-n-Point (PnP) problem (Fischler and Bolles, 1981). For each matched reference point in frame t−1 , we get its 3D position 3D $P_{i}^{\mathrm{cam}}$ in the camera frame using the depth map $D_{t-1}$ , and the camera’s intrinsic matrix K as follows (Equation 2):

$$\;P_{i}^{\mathrm{cam}}=D_{t-1}\left(u_{i}\right)\cdot K^{-1}\cdot {\tilde{u}}_{i}$$ (2)

We estimate the rotation R∈SO(3) and translation $t\in R^{3}$ that minimize the reprojection error of the 3D points of t−1 in the image plane of t , as shown in Equation 3:

$$T^{*}=\underset{R,t}{\mathrm{arg min}}\underset{i}{\sum }\rho \left(\|u_{i}^{t}-\pi \left(K,R,t,P_{i}^{t-1}\right){\|}^{2}\right)$$ (3)

where π(⋅) is the projection function and ρ(⋅) is a robust Huber loss function. This optimization is enclosed in a RANSAC (Random Sample Consensus) loop to reject dynamic objects or outlier reference points, ensuring a robust trajectory estimation.

## 3 Evaluation performance

To evaluate the zero-shot generalization capabilities of our pipeline, we conducted experiments using datasets from a high-fidelity synthetic lunar environment and a real-world Martian analog. This dual-domain strategy allows us to evaluate performance both under extreme lighting conditions such as those found on the moon and in unstructured real-world dynamics such as those found on a Martian analog.

### 3.1 Test dataset

We used two different datasets for the evaluation. First, to evaluate the system’s robustness against the unique visual challenges of the Lunar South Pole, we used the Lunar Segmentation, Navigation and Reconstruction (LuSNAR) dataset (Liu et al., 2024). This dataset was generated using Unreal Engine 4, simulating photorealistic lunar terrain with variable rock distributions and crater densities. It represents high dynamic range lighting conditions typical of lunar poles, creating deep shadows and high-contrast scenes that challenge traditional feature detectors. We processed high-resolution 1024×1024 pixel stereoscopic image pairs converted into monocular sequences. LuSNAR provides perfectly synchronized 6-DoF depth poses and dense depth maps obtained from the simulation engine’s Z-buffer. This allows us to isolate the performance of the visual odometry algorithm by providing ideal scale recovery, separating geometric estimation errors from depth estimation noise.

Second, to evaluate the simulator-real transfer gap, we used the Katwijk Beach Planetary Rover dataset (Hewitt et al., 2018), which was collected at a Martian analog site in the Netherlands. This dataset captures a 1 km traverse along a sandy beach covered with artificial rocks arranged to emulate the size-frequency distribution of Martian landing sites. The terrain is unstructured and sedimentary, presenting visual aliasing challenges similar to those encountered on Mars. The data was captured by a rover platform equipped with stereo cameras and LiDAR. For our experiments, we used the left camera stream to simulate a monocular setup, while employing the available stereo disparity for depth extraction, introducing real sensor noise into the pipeline. The dataset provides centimeter-level accuracy positioning via Real-Time Kinematic (RTK) GPS, enabling accurate evaluation of trajectory drift in the XY plane.

### 3.2 Evaluation metric

To quantify the accuracy of the trajectory estimation, we use the Absolute Trajectory Error (ATE), a standard metric for visual odometry consistent with the KITTI (Geiger et al., 2012) and EuRoC (Burri et al., 2016) benchmarks.

ATE measures the overall consistency of the estimated trajectory by comparing the absolute distances between the estimated poses $P_{\mathrm{est}}$ and the ground truth positions $P_{gt}$ . Given that monocular VO trajectories can have arbitrary global origins, we first align the trajectories using a rigid body transformation S∈SE(3) through the Umeyama algorithm (Umeyama, 1989). The error at time instant i is defined in Equation 4:

$$E_{i}=P_{gt,i}^{-1}\cdot S\cdot P_{\mathrm{est,i}}$$ (4)

We analyze the Root Mean Square Error (RMSE) of the translation component to evaluate the global drift, as shown in Equation 5:

$$\;{\text{RMSE}}_{\mathrm{ATE}}=\sqrt{\frac{1}{N}\overset{N}{\underset{i=1}{\sum }}\|\text{trans}\left(E_{i}\right){\|}^{2}}$$ (5)

## 4 Experimental results

We evaluate the performance of our Zero-Shot Visual Odometry pipeline in two contrasting domains: the high-fidelity synthetic environment of LuSNAR, characterized by extreme illumination contrast typical of the Lunar South Pole, and the unstructured sandy terrain of the Katwijk Martian analog, which introduces real-world sensor noise and sedimentary dynamics. The objective of these experiments is twofold: first, to quantify the global consistency of the estimated trajectory using the Absolute Trajectory Error (ATE); and second, to validate the internal stability of the foundation model-based frontend by analyzing landmark lifetime and tracking persistence. All sequences were processed over 100 frame window, within the memory limit of the available GPU. The evaluated sequences and their characteristics are summarized in Table 1 where the number of logged poses differs per sequence, because a pose is recorded only when the geometric solver produces a valid estimate, and trajectory metrics are computed over the subset of these poses for which a landmark is available. All experiments were conducted on a workstation equipped with an NVIDIA GeForce RTX 4060 GPU.

**TABLE 1:** Summary of the evaluated sequences. The “Poses logged” column refers to the number of frames for which the pipeline produced a logged pose estimate within the 100-frame processing window.

Sequence	Frames processed	Poses logged	Motion pattern
Moon_1 (LuSNAR)	100	69	Forward motion followed by a sharp rotation maneuver
Moon_2 (LuSNAR)	100	40	Forward traversal with sparse landmarks and deep shadows
Moon_3 (LuSNAR)	100	47	Linear traversal in a feature-rich area
Katwijk (analog)	100	52	Continuous linear traversal on sandy terrain

### 4.1 Trajectory accuracy

The LuSNAR dataset serves as a stress test for visual perception. The absence of atmospheric scattering on the Moon creates pitch-black shadows and blinding highlights, conditions that typically cause handcrafted feature extractors (like SIFT or ORB) to fail due to a lack of gradient information in shadowed regions. To assess the consistency of our approach, we analyzed three distinct traverses within the dataset.

**FIGURE 3:**
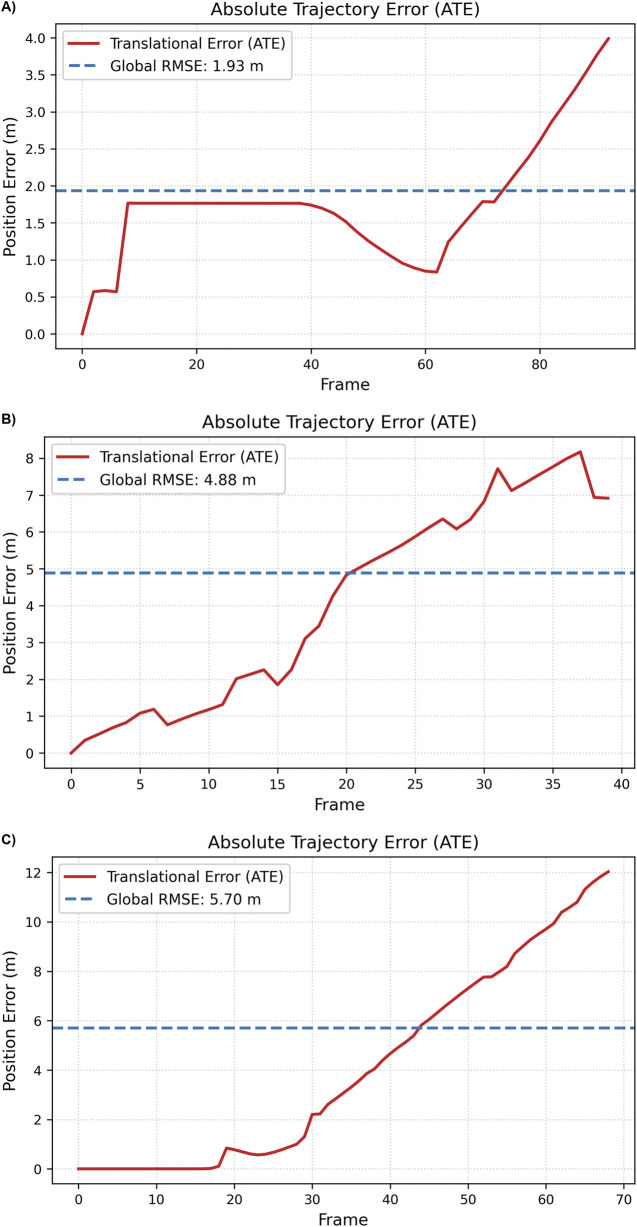
Comparison of trajectory errors (ATE) in the LuSNAR dataset. **(A)** Traverse Moon_3: Scenario with higher stability and an RMSE of 1.93 m. **(B)** Traverse Moon_2: Intermediate behavior with an RMSE of 4.88 m and error accumulation by steps. **(C)** Traverse Moon_1: Highest stress scenario with an RMSE of 5.70 m, showing super-linear drift after a sudden rotation maneuver.

To see the consistency of our approach across different geological and lighting conditions, we expanded this error analysis into three distinct traverses as shown in Figure 3. Moon_3 demonstrated the highest stability, achieving a global RMSE of 1.93 m. The error curve (Figure 3A) shows a rapid initial stabilization, maintaining a flat plateau for most of the traverse. This indicates continuous, highly robust tracking before a final drift spike caused by a sudden loss of scale observability in a featureless region. On the other hand, traverse Moon_2 (Figure 3B) presented a moderate challenge with an RMSE of 4.88 m. The error growth is non-linear, with intermittent steps that suggest partial tracking re-initializations due to sparse landmark distribution most probably caused by severe terrain self-occlusions, deep shadows, or sparse rock distributions that force the data association module to constantly re-initialize tracks. Finally, traverse Moon 1 recorded the highest drift with a global RMSE of 5.70 m. The error profile (Figure 3C) exhibits a linear growth during forward motion (frames 0–40) followed by a super-linear accumulation triggered by a sharp rotation event. This behavior is characteristic of monocular systems where heading errors during turns amplify lateral position drift over distance.

**FIGURE 4:**
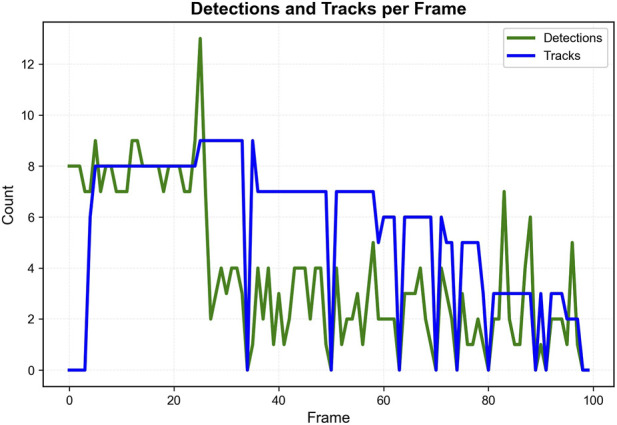
Detection and tracking per frame. The green line indicates raw SAM proposals, while the blue line represents landmarks successfully tracked by DINOv2. The number of tracked landmarks fluctuates over the traverse, illustrating the descriptor’s ability to re-identify landmarks across changing lighting conditions.

To determine whether this drift was caused by a failure in semantic landmark detection or by geometric estimation limitations, we analyzed the tracking statistics (Figure 4). The green line indicates the raw number of rock proposals generated by SAM, while the blue line represents the number of stable tracks successfully associated by DINOv2 across consecutive frames. The system generates a fluctuating number of raw rock detections (green line), peaking at 13 detections in feature-rich frames. This density proves that the Segment Anything Model is robust to lunar lighting conditions; it successfully segments rocks even when they are partially obscured by deep shadows, a task where threshold-based detectors typically fail. The number of stable tracks (blue line) fluctuates over the traverse, with peaks in the range of 7-9 and intermittent drops to lower values in segments where landmarks moved out of view or association failed. Despite these fluctuations, sufficient tracks were maintained during most of the sequence to support continuous pose estimation, suggesting that the DINOv2 descriptors enable re-identification of previously seen landmarks across consecutive frames. This pattern is consistent with track loss caused by a geometric loss of overlap as landmarks move out of the camera field of view.

**FIGURE 5:**
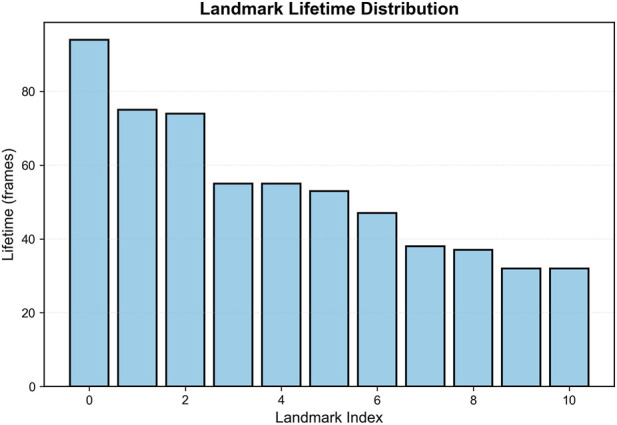
Per-landmark lifetime in LuSNAR. The vertical axis shows the number of consecutive frames each landmark was tracked. Long-lived tracks indicate stable re-identification across changes in viewpoint and scale.

Further evidence of the semantic robustness is provided in Figure 5, which reports the lifetime of each individual landmark, defined as the number of consecutive frames over which it was tracked. Several landmarks remained associated for 60 to 90 frames, indicating that DINOv2 descriptors enable re-identification across significant changes in viewpoint and scale. In a visual odometry context, features with long lifetimes are the most valuable as they reduce scale drift. The fact that DINOv2 descriptors allowed re-identification of rocks over such a long duration—despite the significant change in viewing angle and scale as the rover approached them—validates the view-invariance of our zero-shot approach.

**FIGURE 6:**
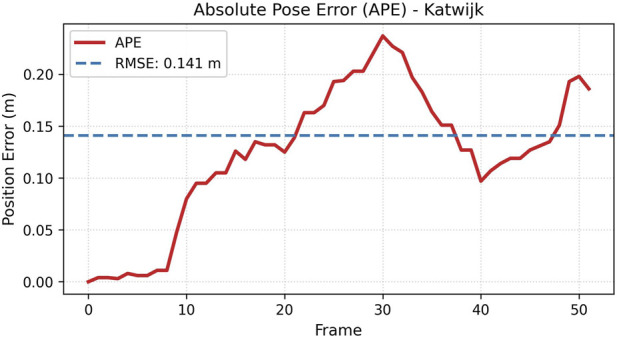
Evolution of the Absolute Trajectory Error (ATE) in the Katwijk path. The system maintains a bounded error with a global RMSE of 0.141 m, validating the pipeline’s accuracy under diffuse lighting conditions.

**FIGURE 7:**
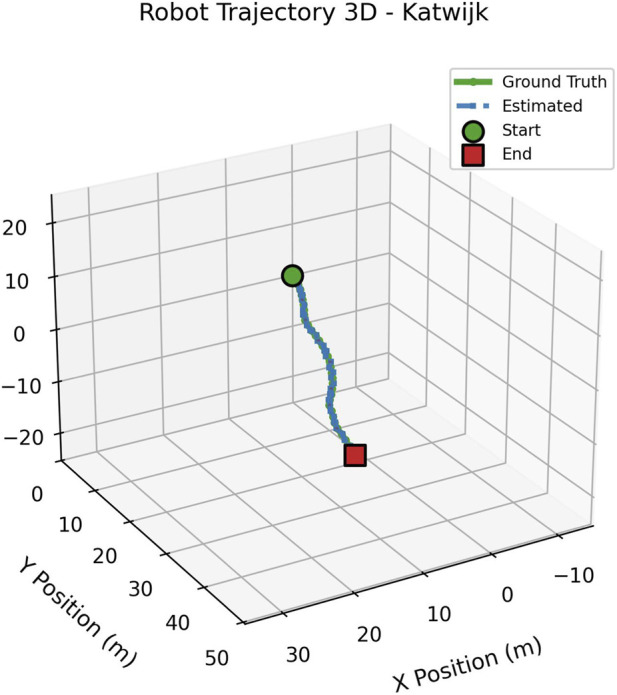
Estimated 3D trajectory vs. Ground Truth at the Katwijk Martian analog. The estimate (blue dashed line) shows high fidelity to the actual trajectory (green solid line) in an unstructured sandy terrain environment.

**FIGURE 8:**
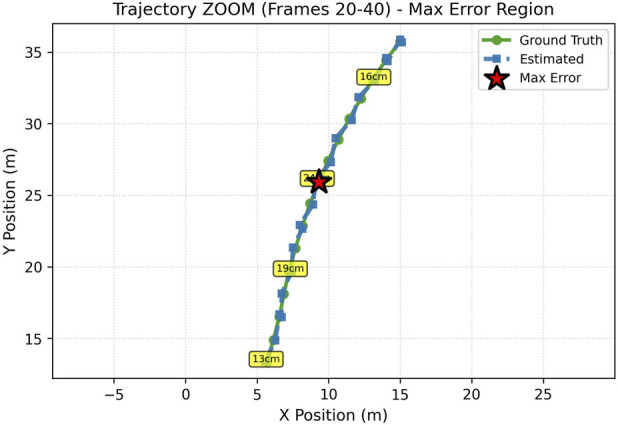
Granular analysis of the region of maximum error (Frames 20–40). The red star marks the point of maximum divergence (24 cm); the remaining annotations (13, 16, 19 cm) report the error at representative frames along the segment. The system’s recovery capacity after overcoming irregularities in the sedimentary dynamics of the terrain is observed.

In contrast to the synthetic domain, the deployment in the real-world Katwijk Martian analog demonstrates the system’s capability when semantic landmarks are complemented by terrain texture. Figure 6 illustrates the evolution of the Absolute Trajectory Error over the traverse achieving a global RMSE of 0.141 m. Figure 7 illustrates the qualitative 3D trajectory, where the estimated path (blue dashed line) adheres closely to the ground truth (green solid line). Unlike in the lunar case, the error distribution does not exhibit a runaway trend. The error remains bounded between 0.10 m and 0.20 m for the entire duration of the experiment. A granular analysis of the region of maximum divergence (Figure 8) shows that the error peaks at around 24 cm before the estimated path returns to the ground truth. Several factors may contribute to the lower error observed in Katwijk compared to the lunar traverses. Among them, the higher frequency textural content of the sandy terrain provides richer contextual cues around each rock, which the DINOv2 descriptor encodes within its receptive field. In contrast, the lunar traverses present feature-sparse regolith between rocks, deep shadows, and sharper rotational maneuvers, all of which are known to be challenging for monocular VO. These results were obtained using the same pre-trained foundation model weights used in the lunar simulation, with no fine-tuning or parameter adjustment between domains. This consistency across two visually distinct environments, a high-contrast lunar scene and a diffuse overcast sandy beach, provides preliminary evidence of cross-domain transfer enabled by the foundation-model priors.

We compare our approach against existing planetary localization methods summarized in Table 2 (Grip et al., 2022; San Martin et al., 2017; Van Diggelen and Enge, 2015). Traditional orbital matching methods, such as Terrain Relative Navigation (TRN) and LunarNav, typically report accuracies in the range of 5.0 m–10.0 m. These methods are visually limited by the resolution of the satellite imagery (typically 0.5–1.0 m per pixel). Our approach achieves 0.14 m accuracy in the Martian analog domain without relying on a prior orbital map. This level of precision is relevant for fine-approach maneuvers, such as aligning a drill with a specific rock or docking with a charging station, tasks where a 5-m error would be catastrophic. While the lunar simulation showed higher drift (5.70 m) on the most challenging traverse, this is attributed to geometric factors such as sharp rotational maneuvers and reduced field-of-view overlap.

**TABLE 2:** Qualitative comparison of representative planetary localization and visual odometry methods.

Method	Modality	Locale	Task type	Requires prior map	Accuracy [m]
GPS (Van Diggelen and Enge, 2015)	Satellite time code	Earth	Global localization	Yes	4.9
Geo-referencing (Parker et al., 2010)	Satellite imagery	Mars	Global localization	Yes	0.5
TRN (Johnson et al., 2007; VanderHook et al., 2022)	Local imagery	Mars	Global localization	Yes	10.0
MAVeN (San Martin et al., 2017; Grip et al., 2022)	Aerial imagery	Mars	VO with aerial scale	No	3.0
LunarNav (Daftry et al., 2023)	Local imagery	Moon	Global localization	Yes	5.0
ShadowNav (Atha et al., 2024)	Local imagery	Moon	Global localization	Yes	1.5
Ours (proposed)	Local imagery	Moon/Mars analog	Semantic landmark-based VO	No	1.93/0.14

The compared approaches differ in terms of sensing modality, task definition, and prior-map dependence. Our method targets local VO, without prior maps, and its accuracy is reported separately for the best lunar traverse and for the Martian analog.

### 4.2 Ablation study

To isolate the contribution of the main components of the proposed pipeline, we conducted an ablation study on the Moon_3 sequence. We selected this sequence as a representative lunar scenario with stable landmark detection and the most favorable trajectory accuracy among the lunar traverses. Restricting the ablation to a single sequence keeps the comparison controlled and computationally tractable, given the high per-frame cost of the SAM backbone (approximately 28 s/frame on the available hardware). We evaluated five components: the solidity threshold of the geometric filter, the type of crop passed to the DINOv2 encoder, the presence of geometric filtering, the DINOv2 backbone, and the segmentation backbone (SAM, SAM2, SAM3). All other components were kept identical to the baseline described in Section 2. The results are summarized in Table 3.

**TABLE 3:** Ablation study on the Moon_3 sequence. RMSE values marked with * are computed over truncated trajectories and are not directly comparable to the full-sequence baseline.

Ablation	Variant	RMSE [m]	Frames	Observation
Baseline	S = 0.85, tight, ViT-S, SAM	1.93	94	References
Solidity	S = 0.5	0.61 *	29	Tracking lost early
Solidity	S = 0.95	—	0	Too strict; no pose
Crop	Masked/tight/expanded	1.93	94	Trajectory unchanged
Geom. Filter	OFF	2.57	43	Worse despite shorter traj
DINOv2	ViT-B/14	0.66 *	30	No gain; 10x load time
Segmenter	SAM2	8.68	98	Worse; higher drift
Segmenter	SAM3	0.33 *	32	Fast (1.2s/fr); tracking lost

All segmentation backbones were run in automatic mode.

#### 4.2.1 Solidity threshold

We varied the solidity threshold of the geometric filter across three values. With a permissive threshold (S = 0.5), the filter admitted lower-quality masks; tracking was lost after 29 frames, producing a truncated trajectory whose apparently lower RMSE (0.61 m) reflects only the initial, low-drift portion of the traverse and is therefore not directly comparable to the full-sequence baseline. With a strict threshold (S = 0.95), almost no masks passed the filter and the system failed to maintain the minimum of four landmarks required for pose estimation, yielding no successful localizations. The baseline value (S = 0.85) sustained tracking across the full 94-frame range, confirming it as an appropriate balance between mask quantity and quality.

#### 4.2.2 Crop context

We compared three crop strategies for the region passed to the DINOv2 encoder: masked crops (only the pixels within the SAM mask, background set to zero), tight bounding-box crops (baseline), and expanded-context crops (bounding box enlarged by a factor of 1.5). A descriptor-level analysis shows that the crop strategy substantially alters the embeddings: the mean cosine similarity between the same landmark’s descriptors was 0.36 (masked vs. tight), 0.30 (masked vs. expanded), and 0.71 (tight vs. expanded). The notably lower similarity of the masked variant indicates that the immediate context surrounding each rock is encoded in the descriptor. Nonetheless, the estimated trajectory was identical across all three crop variants, indicating that data association in this sparse-landmark regime is robust to the choice of crop and that the method behaves as effectively object-level. This ablation could not be reproduced on the texturally richer Katwijk sequence, where sparse rock detections do not provide enough simultaneous landmarks for a controlled comparison.

#### 4.2.3 Geometric filtering

Removing the geometric filtering stage caused the number of accepted detections per frame to increase substantially (from approximately 3–5–32.4), as all SAM masks—including shadows, terrain texture, and non-rock regions—were treated as candidate landmarks. Despite covering a shorter portion of the traverse (43 vs. 94 logged frames), the unfiltered configuration produced a higher trajectory error (2.57 m vs. 1.93 m). Since shorter trajectories typically accumulate less drift, a higher error over a shorter segment indicates that the additional low-quality landmarks actively degrade pose estimation. This confirms that the geometric filtering stage contributes to both tracking stability and accuracy.

#### 4.2.4 DINOv2 backbone

We replaced the DINOv2 ViT-S/14 backbone (384-dimensional descriptors) with the larger ViT-B/14 variant (768-dimensional descriptors). The larger backbone did not improve tracking: the system sustained tracking over a shorter portion of the traverse (frames 8–38) than the ViT-S/14 baseline (frames 5–99), while increasing model loading time roughly tenfold (from approximately 3–4 s to approximately 31 s) and doubling the descriptor dimensionality. This indicates that the smaller ViT-S/14 backbone is sufficient for landmark re-identification in this setting and that descriptor capacity is not the limiting factor for trajectory length.

#### 4.2.5 Segmentation backbone

We compared the original SAM (ViT-B) against SAM2 and SAM3, all in automatic mode, keeping other components identical to the baseline. SAM2 processed the full sequence but produced more detections per frame (7.96) and substantially higher trajectory error (8.68 m vs. 1.93 m) with elevated drift, indicating that its additional detections introduced less stable landmarks. SAM3 processed frames roughly twenty times faster (approximately 1.2 s vs. 28 s per frame) but sustained tracking only up to frame 32; although it continued to produce and match detections (five to six per frame), the temporal tracker could not maintain stable tracks thereafter, falling below the minimum required for pose estimation. Since the core principle of our method is to preserve rocks as persistent landmarks across frames, this loss of track continuity directly undermines pose estimation: SAM3’s detections, while individually valid and fast to compute, proved insufficiently consistent for the long-lived tracking that our approach requires. Overall, the original SAM backbone provided the most stable tracking across the full traverse, while the newer variants either degraded accuracy (SAM2) or compromised tracking continuity (SAM3) in this configuration. SAM3’s markedly lower per-frame cost nonetheless suggests a promising direction if combined with a tracker adapted to its detection characteristics.

#### 4.2.6 Runtime and computational cost

We measured the runtime of the pipeline on the workstation GPU (NVIDIA RTX 4060). Model loading takes approximately 3–4 s with the ViT-S/14 backbone (rising to approximately 31 s with ViT-B/14) and occurs once at startup. With the original SAM backbone, the dominant cost is the mask generation stage, requiring approximately 28–33 s per frame, whereas DINOv2 descriptor extraction is negligible (on the order of 0.01 s per landmark), giving an overall throughput of approximately 0.03–0.04 frames per second. Notably, SAM3 in automatic mode reduced the per-frame cost to approximately 1.2 s, roughly a twentyfold speed-up, although at the cost of tracking continuity as described above. These measurements identify the segmentation backbone as the principal computational bottleneck and explain the memory constraint reported in this section, where the cumulative landmark database and descriptor cache exceed the available GPU memory.

Across all ablation experiments, the baseline configuration sustained tracking over the longest portion of the traverse (94 logged frames), whereas every alternative configuration lost tracking earlier or produced higher error. This consistent pattern indicates that the baseline parameter choices are well suited to maintaining stable landmark association in the lunar domain.

## 5 Prospective studies

The perception frontend relies on the SAM backbone, which dominates the per-frame runtime (approximately 28–33 s per frame on an NVIDIA RTX 4060) and constrains the sequence length: beyond approximately 110 frames. The combined memory footprint of the cumulative landmark database and the DINOv2 descriptor cache exceeds the available GPU memory. DINOv2 descriptor extraction, by contrast, is negligible (on the order of 0.01 s per landmark) and is not a bottleneck. Extending the approach to longer trajectories and onboard deployment will require lighter foundation-model variants (e.g., FastSAM, MobileSAM, or distilled SAM and DINOv2 models) together with external memory-management strategies. Our ablation with SAM3 in automatic mode is encouraging in this regard, as it reduced the per-frame cost roughly twentyfold (to approximately 1.2 s); however, its detections did not sustain stable long-term tracking in the current pipeline, indicating that a tracker adapted to its detection characteristics would be needed to exploit this speed-up.

The geometric backend depends on an external depth source (the simulator Z-buffer in LuSNAR, and stereo disparity in Katwijk), and the sensitivity of the pipeline to depth noise has not been quantified in this work. In future work we will evaluate the impact of depth uncertainty on the PnP solution and explore the integration of inertial measurements (IMU) to mitigate the scale drift observed in the longer monocular lunar traverses.

The current evaluation covers four 100-frame segments across two datasets (LuSNAR and Katwijk Beach). The results indicate the potential of foundation-model-based semantic landmarks for zero-shot visual odometry in planetary-like environments, but stronger claims about generalization will require evaluation on longer trajectories and across more diverse terrains, illumination conditions, and motion patterns. Datasets such as MADMAX and POLAR offer additional planetary-analog conditions that could be explored in future iterations.

This study evaluates a frame-to-frame visual odometry frontend and does not include a comparison with full visual SLAM systems such as ORB-SLAM3 or DROID-SLAM. These systems address tasks beyond frame-to-frame odometry, including loop closure, global mapping, and bundle adjustment, and typically rely on stereo or inertial input in their reported configurations. A controlled benchmark on the same planetary sequences, ideally integrating the proposed semantic-landmark frontend as a replacement for low-level keypoints within such systems, would help position the approach within the broader VO/SLAM landscape and is an important direction for future work.

The ablation study was conducted on a single representative lunar sequence (Moon_3) due to the high per-frame cost of the SAM backbone. Extending the ablation to additional sequences and domains, and to further components such as alternative descriptors (e.g., SIFT, ORB, SuperPoint) and detection thresholds, would provide a more complete characterization of which components contribute most to performance. In particular, the contribution of contextual information around landmarks, which we observed at the descriptor level but not at the trajectory level in the sparse lunar regime, warrants evaluation in the texturally richer Katwijk domain, where sparse rock detection currently prevents a controlled comparison.

Taken together, these limitations define a roadmap toward a lighter, more thoroughly validated, and more broadly comparable system for semantic-landmark-based visual odometry in unstructured planetary environments.

## 6 Conclusion

In this study, we presented a zero-shot semantic landmark-based visual odometry framework that uses foundation models for landmark detection and description in unstructured planetary environments. By evaluating the system on the LuSNAR and the Katwijk Martian analog datasets, we observed that the proposed pipeline allows to estimate rover trajectories without any domain-specific fine-tuning, suggesting that foundation models offer a promising alternative to low-level feature tracking for planetary VO. We also proposed an adaptive geometric filtering strategy combining the Segment Anything Model and DINOv2. This strategy is designed to isolate stable geological features, taking into account the challenges of extreme illumination and unstructured textures. Our cross-domain evaluation and multi-traverse analysis exposed an observed association between landmark semantic stability and trajectory consistency, achieving a sub-meter accuracy with an RMSE of 0.141 m in the real-world analog scenario. In the lunar context, the best RMSE performance shows 1.93 m and it depends heavily on the local density of available landmarks and the presence of sharp rotational maneuvers. We also carried out ablation studies, which confirmed the contribution of the geometric filtering stage and the sufficiency of the lightweight DINOv2 backbone, while identifying the segmentation model as the main computational bottleneck.

We propose to conduct complementary tests to verify the real-time operational feasibility of the proposed pipeline through simulation-based evaluations, incorporating optimized architectures like FastSAM. Additionally, we intend to couple the visual estimation with inertial measurements (IMU) to mitigate the scale drift observed in long-distance monocular traverses.

## Data Availability

Publicly available datasets were analyzed in this study. This data can be found here: The LuSNAR dataset can be found on GitHub at https://github.com/zqyu9/LuSNAR-dataset. The Katwijk Beach dataset is available via the ESA Robotics Datasets at https://roboshare.esa.int/datasets/index.php/katwijk-beach-planetary-rover-dataset/.
